# The Patent Letheon—Morton and Jackson’s Specification

**Published:** 1847-04

**Authors:** 


12. *The Patent Letheon—Jackson and Morton's Specification.*

[Communicated for the Boston Med. and Surg. Jour.]

It has been repeatedly said that Dr. Jackson is not concerned in the patent for the Letheon; that Mr. Morton alone has taken out the letters patent, and that whatever interest Dr. J. may have in it, arises out of some private contract between them. But it now appears that Dr. J. is really one of the proprietors—that the patent is issued in favor of Morton and Jackson conjointly.

The question has been asked, probably by every member of the profession, "what is patented?" I put the question, the other day, to a gentleman in Boston, who ought to know, and he replied, "The inhalation of the letheon by means of a valvular apparatus." This answer is far from satisfactory, for the same effect may be produced by inhalation of the vapor, without any valvular apparatus at all. If the "apparatus" be an essential part of the patent, the use of a different apparatus would enable any one to evade the penalty of the law. Have they patented the production of insensibility to pain by the inhalation of etheric vapor? No. A physician may administer the vapor, and produce insensibility to pain with or without the valvular apparatus, without infringing upon the patent. He may administer it for the headache, the heartache, or the bellyache, for tic-douloureux, asthma, or hysterics, and the patent will not reach him. Indeed, I am not quite sure that the patent will reach him if he uses the vapor in reducing dislocation or hernia, or in any operation in which "the knife or other instrument of operation of a surgeon" is not used.

What, then, is the precise thing patented? I answer, the combining with surgical operations, the application of ether, or the vapor thereof. This is the whole thing. The use of it in the practice of medicine, by inhalation, is not patented, nor in surgery even, except when connected with operations. They claim the right to use an old and well-known remedy to produce a given result, in the treatmənt of certain cases. The principle, then, is, that a member of the profession, if he discover that a certain effect may be produced by any remedy or agent in common use, when used in a specified manner, in a certain case or class of cases, which had not (to his knowledge) been previously produced by said remedy or agent, he may secure to himself, by patent, the use of said article for producing this specified effect. For instance, should I discover that tinct. digitalis would cure Dixon Lewis, and others similarly affected, of excessive obesity, as it probably would, I might patent the use of tinct. dig. in such cases. If I discover that hydriod. potassæ, applied in a particular way, will cure dry scab or scurfy eruptions of the skin and scalp, may patent this particular use of it, in this class of cases, and require my brethren to pay me a stipulated sum, or a certain per cent. of the fees they may receive, for the right to use it in such cases. Should I discover that tinct. cayenne pepper and tinct. opii, combined in certain proportions, will cure the cholera, I may claim the sole right to use them in cases of cholera, however many persons may be dying around me for the want of them. If some *Yankee* were now in Bagdad, with a few gallons of these tinctures, with their use secured to him by a patent, would not he coin money?

The use of a known remedy to produce a particular effect in any given branch of professional practice, or in the treatment of a given class of cases, is the principle involved. This, so far as I can discover, from a careful examination of the specification, is the exact principle implied in it. As to the rectitude of this principle, professionally, socially, or morally, I say nothing. Each one can judge for himself. I believe the illustrations I have used above are correct and appropriate—that is, if surgery and the practice of medicine are parts of one and the same profession. If surgery is a mere mechanical operation, and is to take its place in the same category as other operations in mechanics, then the case is altered. Success in the mechanic arts depends, not only upon the skill with which their processes are accomplished, but often upon their processes themselves, and when a man invents a process by which the same result can be accomplished better than before, he is permitted by common consent, to enjoy the benefit of his invention for a limited time. If surgery puts in the same claim for its inventions, let it be divorced from the liberal professions—from the "humanities," and hang out before its office doors, as in the days of Ben Jonson, a staff wound with a red tape, as a *sign* that "surgery is done here." We all know the origin of the barber's pole; and, Mr. Editor, there is a more close connection between surgery and barbery, than one would at first imagine. Many of the operations of *surgery* are *barbarous*, and the operations of *barbery* are often *surgical*. Indeed, many a poor wight would consider it no small alleviation of one of the miseries of human life could he inhale the letheon before submitting to the most common operations of barbery. Mem.—Barbers may use the letheon without infringing upon the patent. With the above remarks, which have extended much farther than I intended, I send you a copy of the specification, which has recently come into my hands, thinking it will gratify the curiosity of many of your brethren. Yours, S.

*March*, 1847.

"*The United States Patent Office.*—To all persons to whom these presents shall come, greeting: This is to certify, that the annexed is a true copy upon the records of this office, of the specification of Jackson and Morton's Letters Patent, dated 12th Nov., 1846.

"In testimony whereof, I, Edmund Burke, Commissioner of Patents have caused the seal of the Patent Office to be hereunto affixed, this twelfth day of February, in the year of our Lord one thousand eight hundred and forty seven, and of the Independence of the Unithd States the seventy-first.

"EDMUND BURKE."

*The Schedule referred to in these Letters Patent, and making Part of the same.*

To all persons to whom these presents shall come: Be it known, that we, Charles T. Jackson and William T. G. Morton, of Boston, in the county of Suffolk, and State of Massachusetts, have invented or discovered a new and useful improvement in surgical operations, such as are usually attended with more or less pain and suffering, without any or very little pain to, or muscular action of persons who undergo the same; and we do hereby declare that the following is a full and exact description of said invention or discovery.

It is well known to chemists that when alcohol is submitted to distillation with certain acids, peculiar compounds termed ethers, are formed; each of which is usually distinguished by the name of the acid employed in its preparation. It has also been known that the vapors of some, if not all these chemical distillations, particularly those of sulphuric ether, when breathed or introduced into the lungs of an animal, have produced a peculiar effect on its nervous system; one which has been supposed to be analagous to what is usually termed intoxication. It has never (to our knowledge) been known until our discovery, that the inhalation of such vapors (particularly those of sulphuric ether) would produce insensibility to pain, or such a state of quiet of nervous action as to render a person or animal incapable to a great extent, if not entirely, of experiencing pain while under the action of the knife, or other instrument of operation of a surgeon, calculated to produce pain. This is our discovery, and the combining it with or applying it to any operation of surgery, for the purpose of alleviating animal suffering, as well as of enabling a surgeon to conduct his operations with little or no struggling or muscular action of the patient, and with more certainty of success, constitutes our invention. The nervous quiet and insensibility to pain produced on a person is generally of short duration; the degree or extent of it, or time which it lasts, depends on the amount of ethereal vapor received into the system, and the constiutional character of the person to whom it is administered. Practice will soon acquaint an experienced surgeon with the amount of etheric vapor to be administered to persons for the accomplishment of the surgical operation or operations required in their respective cases. For the extraction of a tooth the individual may be thrown into the insensible state, generally speaking only a few minutes. For the removal of a tumor or the peformance of the amputation of a limb, it is necessary to regulate the amount of vapor inhaled, to the time required to complete the operation. Various modes may be adopted for conveying the etheric vapor into the lungs. A very simple one is to saturate a piece of cloth or sponge with sulphuric ether, and place it to the nostrils or mouth so that the person may inhale the vapors. A more effective one is to take a glass or other proper vessel like a common bottle or flask. Place in it a sponge saturated with sulpheric ether. Let there be a hole made through the side of the vessel for the admission of atmospheric air (which (*hole*) may or may not be provided with a valve opening downwards, or so as to allow air to pass into the vessel) a valve on the outside of the neck opening upwards, and another valve in the neck, and between that last mentioned and the body of the vessel or flask, which latter valve in the neck should open towards the mouth of the neck or bottle. The extremity of the neck is to be placed in the mouth of the patient, and his nostrils stopped or closed in such manner as to cause him to inhale air through the bottle, and to exhale it through the neck. The air thus breathed, by passing in contact with the sponge will be charged with the etheric vapors, which will be conveyed by it into the lungs of the patient. This will soon produce the state of insensibility or nervous quiet required.

In order to render the ether agreeable to various persons we often combine it with one or more essential oils, having pleasant perfumes. This may be effected by mixing the ether and essential oil, and washing the mixture in water. The impurities will subside, and the ether impregnated with the perfume will rise to the top of the water. We sometimes combine a narcotic preparation, such as opium or morphine with the ether. This may be done by many ways known to chemists, by which a combination of narcotic vapors may be produced.

After a person has been put into the state of insensibility, as above described, a surgical operation may be performed upon him, without, so far as repeated experiments have proved, giving to him any apparent or real pain, or so little in comparison to that produced by the usual process of conducting surgical operations, as to be scarcely noticeable. There is very nearly if not entire absence of all pain. Immediately or soon after the operation is completed, a restoration of the patient to his usual feeling takes place, without, generally speaking, his having been sensible of the performance of the operation.

From the experiments we have made we are led to prefer the vapors of sulphuric ether to those of muriatic or other kinds of ether, but any such may be employed which will properly produce the state of insensibility without any injurious consequences to the patient.

We are fully aware that narcotics have been administered to patients undergoing surgical operations, and as we believe always by introducing them into the *stomach.* This we consider in no respect to embody our invention, as we operate through the *lungs and air passages*, and the effects produced upon the patient are entirely or so far different as to render the one very little, while the other is of immense utility. The consequences of the change are very considerable, as an immense amount of human or animal suffering can be prevented by the application of our discovery.

What we claim as our invention is the hereinbefore described means by which we are enabled to effect the above highly important improvement in surgical operations, viz.: by combining therewith the application of ether or the vapor thereof substantially as above specified.

In testimony whereof we have hereunto set our signature this twenty-seventh day of October, A. D. 1846.

Charles T. Jackson,

Wm. T. G. Morton.

Witnesses,

*R. H. Eddy*,

*W. H. Leighton.*

